# Use of intravenous and intramuscular immunoglobulin in the practice of treatment for purulent and septic deaths in newborns

**DOI:** 10.1186/cc11800

**Published:** 2012-11-14

**Authors:** G Khanes, L Nazarchuk, I Maksakova, O Bakaieva

**Affiliations:** 1 Ukrainian National Paediatric Hospital OkhMatDyt, Kyiv, Ukraine; 2 Ukrainian Research Institute of Hematology and Transfusiology, Kyiv, Ukraine

## Background

The investigations of A Fedorovska and L Nazarchuk (1995 to 2000) showed the necessity to introduce specific antigens of G class for the treatment of sepsis in immunodeficiency patients. We refer newborns to such patients. Effective treatment of sepsis in newborns and children of a young age group is impossible without evaluation of immunocompetent systems.

## Methods

We successfully used intravenous normal human immunoglobulin *Bioven mono *when extracting anti-staphylococcus, anti-proteus and anti-pseudomonas antibodies in 150 newborns with postoperative and bone and joint sepsis. This immunoglobulin has international certification. During the last 10 years the main problem for treatment of postoperative sepsis in newborns is the treatment of pseudomonade infection. Regarding that traditional preparations have an insignificant titer of anti-pseudomonade antibodies, we used new anti-pseudomonade immunoglobulin received from specific plasma for 37 newborns and children under 1 year old. There were 20 patients having sepsis with esophageal atresia, two patients with angiocardiogenic sepsis, five with laryngotracheostenosis, five with bone and joint sepsis, and five after polytrauma with injury of pelvis bones and urinary bladder. In these 37 patients we used a new method for introduction of immunoglobulin in combination with fresh frozen plasma (patented).

## Results

Use of *Bioven *in combination with antibacterial therapy allowed treatment of up to 90% of infants with severe sepsis. The use of new anti-pseudomonade immunoglobulin allowed recovery of the susceptibility of pathogenic flora to antibiotics after triple introduction of specific immunoglobulin, to raise phagocytal response and thus to gain positive results in 35 patients.

## Conclusion

The experience of immune system correction in newborns with postoperative and bone and joint sepsis allow one to consider this method a leading one in clinic practice. The use of specific immune preparations increases complementary activity of blood and antibiotic susceptibility of pathogenic microflora.

